# Medical Opinion and Movement

**Published:** 1906-10-06

**Authors:** 


### Medical Opinion and Movement.

The annual service of the Guild of St. Luke will be held in St. Paul's Cathedral on Wednesday, October 17, at 7.30 p.m.

Sir Victor Horsley has resigned his seat on the General Medical Council to avoid the inconvenience of a separate election next year.

Mr. H. S. Wellcome's liberality in publishing the report of the laboratories of the Gordon Memorial College at Khartoum has elicited one interesting fact. Through the services of six men under an English inspector for an outlay of less than £100 per annum, Khartoum is now kept practically free from malaria and the perpetual torment of dangerous mosquitoes.

It is a curious fact, as showing different points of view in public and even medical opinion, that in Victoria, where an Amending Medical Practitioners Bill has been read a second time, strong opposition was raised against it by the Homœopathic Hospital, its managers and supporters. The new Act provides for a five years' medical course and the homœopathic hospital people are opposing this enactment on the ground that if it be passed they will have difficulty in procuring a medical staff. The reason for this is that this hospital declares that America is the only place it can draw upon for homœopathic practitioners, and that the course there is much less than five years. Such is homœopathy in the Australian Colonies.

We recently dealt with goats' milk for infants. Lieutenant-Colonel Giles in "Climate and Health in Hot Countries" gives as his experience that goats, being naturally clean feeders, their milk usually agrees excellently with infants, and its flavour is preferred by many to cows' milk. Goats' milk often agrees with children where cows' milk fails; but asses' milk, in Colonel Giles's opinion, is superior to both, and may be regarded as the best substitute for an infant's natural food. Colonel Giles points out that in India, when pressed by hunger, there is no fouler feeder than the cow, and it is a dismal fact that in the polity of an Indian village the cattle rival the pigs in their efficiency as scavengers. This is not generally appreciated.

A writer in the *Lancet* has calculated the face value of the patent medicines sold in one year as represented by the value of the revenue stamps. Nearly forty million packages of patent medicine were sold with a face value of over two and three-quarter million pounds sterling. These figures indicate that on the average every inhabitant of Great Britain consumes one packet of patent medicine every year. Nothing could testify more clearly to the average lack of intelligence amongst the people, or show their reckless disregard to their individual interests in matters of health. The Government makes a large revenue by permitting the sale of these patent remedies; but seeing that China has determined to prohibit the sale of opium, may we not hope that a British Government will soon arise to protect the British public by restricting the sale of harmful and worthless patent medicines?

Ought practitioners to charge clergymen fees, and what fees should a dentist demand from the practitioner of medicine? Our view is that every practitioner and every dentist is entitled to his fee, whoever the patient may be. If a practitioner chooses to give free attendance to any patient that is his affair, but there is no obligation on any member of the profession to do this in any case. A dentist has to give not only his experience, knowledge, and mechanical skill in treating his patient, but he has to incur expense in providing the necessary apparatus; and there is certainly no obligation upon any dentist in such circumstances to give free attendance to a medical practitioner. The dentist, like the practitioner, is a free agent, however, and may make a present of his services at his discretion. But free service in all these cases must, in fact, be a present which will place the recipient under an obligation to the giver. Generally it is bad business for practitioners and dentists to render free service at all unless in very exceptional circumstances.

Dr. Crothers, of Hartford, Connecticut, is very hard on the moderate drinker. He contends that "the moderate drinker is the subject of a pronounced psychosis, and fully as insane as the periodic inebriate. Recent laboratory research with instruments of precision, sustained by exact clinical studies, prove this assertion to be absolutely correct. The common theory that alcohol in small doses may be taken regularly without harm is not true. Instruments show that alcohol in small doses depresses the sight, hearing, taste, smell, and also the touch. It lowers the functional activity of the organs, and disturbs the rhythmic circulation of the blood. Clinical studies yield evidence showing that alcohol in small doses lowers resistance to microbic invasions and acute infectious diseases, whilst it devitalises the forces of both mind and body." Moderate drinkers may further be interested to learn, according to Dr. Hobbs Guelph, that the insanity of inebriety develops in three stages: (1) Defective will power, or insanity of the ego; (2) delusional stage, with delusions of suspicion; (3) dementia. Moderate drinkers develop mental inconsistency, irregularities, and other defects. Such changes may not be called insanity, but they are clearly stages of degeneration.

